# Hampering Herpesviruses HHV-1 and HHV-2 Infection by Extract of *Ginkgo biloba* (EGb) and Its Phytochemical Constituents

**DOI:** 10.3389/fmicb.2019.02367

**Published:** 2019-10-15

**Authors:** Marta Sochocka, Maciej Sobczyński, Michał Ochnik, Katarzyna Zwolińska, Jerzy Leszek

**Affiliations:** ^1^Laboratory of Virology, Hirszfeld Institute of Immunology and Experimental Therapy, Polish Academy of Sciences, Wrocław, Poland; ^2^Department of Genomics, Faculty of Biotechnology, University of Wrocław, Wrocław, Poland; ^3^Department of Psychiatry, Wrocław Medical University, Wrocław, Poland

**Keywords:** extract of *Ginkgo biloba* (EGb), isorhamnetin, HHV-1, HHV-2, antiviral activity, viral particles trapping

## Abstract

Despite the availability of several anti-herpesviral agents, it should be emphasized that the need for new inhibitors is highly encouraged due to the increasing resistant viral strains as well as complications linked with periods of recurring viral replication and reactivation of latent herpes infection. Extract of *Ginkgo biloba* (EGb) is a common phytotherapeutics around the world with health benefits. Limited studies, however, have addressed the potential antiviral activities of EGb, including herpesviruses such as *Human alphaherpesvirus 1* (HHV-1) and *Human alphaherpesvirus 2* (HHV-2). We evaluated the antiviral activity of EGb and its phytochemical constituents: flavonoids and terpenes against HHV-1 and HHV-2. Pretreatment of the herpesviruses with EGb prior to infection of cells produced a remarkable anti-HHV-1 and anti-HHV-2 activity. The extract affected the viruses before adsorption to cell surface at non-cytotoxic concentrations. In this work, through a comprehensive anti-HHV-1 and anti-HHV-2 activity study, it was revealed that flavonoids, especially isorhamnetin, are responsible for the antiviral activity of EGb. Such activity was absent in quercetin and kaempferol. However, EGb showed the most potent antiviral potency compared to isorhamnetin. EGb could augment current therapies for herpes labialis and genital herpes. Moreover, the potential use of EGb in multidrug therapy with synthetic anti-herpes compounds might be considered.

## Introduction

Viral infections are still a key public health problem and a significant cause of many epidemic diseases worldwide. Vaccines and antiviral therapies are effective tools to control viral infections. There are many vaccines for viral diseases like measles, flu, or rubella. On the other hand, antiviral drugs play an important role against viral pathogens for which protective vaccines are unavailable, such as *Human alphaherpesvirus 1* (HHV-1) (Birkmann and Zimmermann, 2016). HHV-1 and *Human alphaherpesvirus 2* (HHV-2) belong to the most common worldwide infections of humans, producing lifelong infection. It is estimated that among people under the age of 50, 3.7 billion are infected with HHV-1 and about 417 million suffer of HHV-2 infection (Rechenchoski et al., 2017). After primary infection, through the mucous membrane or damaged skin, viruses become latent and exist in the trigeminal ganglia (HHV-1) or lumbar-sacral ganglia (HHV-2) (Boldogh et al., 1996). Several factors, such as UV, stress, injury, immune impairment, or immunosenescence lead to virus reactivation in the place of the initial infection, causing pain, and skin ailments (blisters filled with serous fluid with active virions). Skin lesions are related the oral cavity, lips, hands, or genital organs (Rechenchoski et al., 2017). In extreme situations, reactivation of HHV-1 can lead to serious complications such as encephalitis and meningitis (Tyler, 2004). It was shown that HHV-1 can enter the brain in older age and establish a latent infection (Itzhaki, 2018). Recurrent reactivation, in turn, causes the activation of neuroinflammatory process and herpes simplex encephalitis (HSE). It is suggested that incidents of mild HSE may lead to Alzheimer’s disease (AD) (Itzhaki and Tabet, 2017). Several antivirals are available for HHV-1 and HHV-2 infections, such as acyclovir, valacyclovir cydofovir, or penciclovir (James and Prichard, 2014). These preparations, however, have shortcomings such as a relatively fast emergence of resistance in the immunocompromised patients and risk of generating drug-resistant infections. Moreover, drug toxic side effects were also reported (James and Prichard, 2014). Therefore, it is necessary to develop and expand the scope of antivirals. We believe that the most promising are preparations of a natural origin as an alternative to commercially available synthetic preparations.

Plants are a valuable source of substances with antiviral activities as a defense against invading pathogens. Extract of *Ginkgo biloba* (EGb) dried green leaves belongs to the most common phytotherapeutics around the world (Isah, 2015). EGb has a long history of use in the treatment of heart and lung diseases, and is also known to improve memory function (Mohanta et al., 2014). EGb is commercially available as a one of the five top-selling herbal supplements (Ude et al., 2013). Flavonoids, terpenes, and organic acids are the main components of EGb. The standardized extract contains flavonoids expressed as flavone glycosides (22–27%), e.g., kaempferol, quercetin, isorhamnetin, 2.8–3.4% terpene trilactones (ginkgolides A, B, C), 2.6–3.2% bilobalide and less than 5 ppm ginkgolic acids due to its cytotoxic potency (Mohanta et al., 2014; Isah, 2015). The scientifically proven effect of EGb is related to the beneficial influence on the CNS and cardiovascular systems. It is widely used in various neurodegenerative diseases such as Parkinson’s Disease or AD (Zuo et al., 2017). In the abundant scientific data indicating the therapeutic properties of EGb, there is no information about the use of EGb in the prevention and treatment of viral diseases. Limited scientific information indicated the potential antiviral activities of EGb or *G. biloba* compounds for several viruses. It was showed that polysaccharides from *G. biloba* fruit exocarp exhibit activity against PEDV (*Porcine epidemic diarrhea virus*) (Lee et al., 2015), and ginkgolic acid from *G. biloba* inhibited HIV (*Human immunodeficiency virus*) protease (Lü et al., 2012). Nanoemulsion with polyphenols isolated from *G. biloba* leaves and EGb exhibit anti-influenza H1N1 and H3N2 activity as well as anti-HBV (*Hepatitis B virus*) (Haruyama and Nagata, 2013; Wang et al., 2015).

In the present *in vitro* studies, we investigated the antiviral activity of standardized EGb against HHV-1 and HHV-2. In addition, we have analyzed the effectiveness of EGb and isorhamnetin in diminishing HHV-1 and HHV-2 infectivity.

## Materials and Methods

### Viruses and Cell Line

*Human alphaherpesvirus 1*; MacIntyre strain, *Herpesviridae* (HHV-1, ATTC VR-539); *Human alphaherpesvirus 2*; MS strain, *Herpesviridae* (HHV-2, ATTC VR-540). HHV-1 and HHV-2 were grown and titrated in A549 cells. The titer was expressed with reference to the TCID_50_ (tissue culture infectious dose) value, based on the cytopathic effects (CPEs) caused by this virus in approximately 50% of infected cells.

A549 (ATCC CCL 185), a human epithelial-like cell line, was maintained in Dulbecco’s Modified Eagle Medium (DMEM) (HIIET PAS, Wrocław, Poland) with antibiotics (100 U/mL penicillin and 100 μg/mL streptomycin), 2 mM L-glutamine, and 10% fetal bovine serum (FBS) (all from Sigma-Aldrich, United States).

### Extract of *G. biloba* (EGb)

Standardized dry extract from *G. biloba* leaves (GINKGONIS EXTRACTUM SICCUM RAFFINATUM ET QUANTIFICATUM PH. EUR. [European Pharmacopoeia]) provided by Martin Bauer Group, Finzelberg GmbH & Co. KG, Andernach, Germany was investigated. EGb is a dry extract from *Ginkgo biloba* leaves. The extract is adjusted to 22.0–27.0% ginkgo flavonoids calculated as ginkgo flavone glycosides, and 5.0–7.0% terpene lactones consisting of 2.8–3.4% ginkgolides A, B, C and 2.6–3.2% bilobalide, and contains less than 5 ppm ginkgolic acids. For a detailed description (HPLC results, microbiological examination) [see section “EGb Characterization” (Supplementary File-Table 1)].

#### EGb Solution

Before each experiment, EGb was dissolved in dimethyl sulfoxide (DMSO) at a primary concentration of 20 mg/ml and mixed thoroughly until complete dissolution. Next, serial concentration of EGb (25–1000 μg/ml) in DMEM 2% FBS was prepared for experiments.

### *G. biloba* Flavonoids Mix

A mix of flavonoids from *G. biloba*: kaempferol, quercetin, and isorhamnetin (100 μg/ml each component in methanol) was obtained from Sigma-Aldrich (St. Louis, MO, United States). Serial concentrations (6–25 μg/ml) were prepared in DMEM 2% FBS for experiments. Methanol (25% and below) does not cause viral inactivation (control). Final concentrations of methanol in tested cells were ≤ 2.5% (non-toxic concentration, control).

### *G. biloba* Terpene Lactones Mix

A mix of terpene lactones from *G. biloba*: bilobalide, ginkgolide A, ginkgolide B, ginkgolide C, ginkgolide J (100 μg/mL each component in acetonitrile) was obtained from Sigma-Aldrich. Serial concentrations (1.5–9 μg/ml) were prepared in DMEM 2% FBS for experiments. Acetonitrile (9% and below) does not cause viral inactivation (control). Final concentrations of acetonitrile in tested cells were ≤ 0.9% (non-toxic concentration, control).

### Flavonoids

Isorhamnetin (3′-methoxy-3,4′,5,7-tetrahydroxyflavone, 3′- methylquercetin) ≥ 95.0% (HPLC); Kaempferol (3,4′,5,7- tetrahydroxyflavone, 3,5,7-trihydroxy-2-(4-hydroxyphenyl)-4H-1-benzopyran-4-one) ≥ 97.0% (HPLC); Quercetin (2-(3,4-dihydroxyphenyl)-3,5,7-trihydroxy-4H-1-benzopyran-4- one, 3,3′,4′,5,6-pentahydroxyflavone) ≥ 95% (HPLC); all obtained from Sigma-Aldrich. Before each experiment, the tested compounds were dissolved in DMSO at primary concentrations, respectively: 5, 25, and 10 mg/ml and mixed thoroughly until complete dissolution. Next, dilution to 1 mg/ml was maintained in methanol. Serial concentrations of each compound (6–25 μg/ml) in DMEM 2% FBS were prepared for experiments. The final concentration of DMSO in tested cells was < 1%. Methanol (25% and below) does not cause viral inactivation (control). Final concentrations of methanol in tested cells were ≤ 2.5% (non-toxic concentration, control).

### Luminescent Cell Viability Assay

CellTiter-Glo^®^ Luminescent Cell Viability Assay (Promega, Poland) was used to determine the number of viable cells. 24-h cultures of A549 cell line were treated with several concentrations of EGb (25–1000 μg/ml) and incubated for 72 h at 37°C. Next CellTiter-Glo assay was performed as previously described (Creţu et al., 2017). Control were cells treated only with culture medium DMEM 2% FBS.

### CTEs Cell Viability Assay

24-h cultures of A549 cell line were treated with several concentrations of EGb (25–1000 μg/ml) and incubated for 72 h at 37°C. Morphological changes of the cells (cytotoxic effects, CTE) were observed every day under inverted microscope and cell viability was calculated based on CTEs, where, 0 - lack of cytotoxic effects, 1- CTEs in 25% of cells, 2 - CTEs in 50% of cells, 3 - CTEs in 75% of cells, and 4 – 100% of the cells affected with CTEs. Control were cells treated only with culture medium DMEM 2% FBS.

### Early Viral Entry Assay (Inactivation Assay)

Inhibition of HHV-1 and HHV-2 replication was examined by early viral entry assay (inactivation assay). Serial concentrations of EGb, *G. biloba* flavonoids mix, *G. biloba* terpene lactones mix, isorhamnetin, kaempferol or quercetin were incubated with HHV-1 or HHV-2 for 0,5 h (‘Short-Term’), 1 and 2 h (‘Long-Term’) at room temperature. Next viruses-tested compounds mixtures were diluted (logarithmic scale) and adsorbed to A549 cells at density 1.8 × 10^5^ cells/ml in 96-well plates. Plates were incubated in 37°C/5% CO_2._ After 3 days of incubation viral CPEs were observed and evaluated under inverted microscope, where, 0 - lack of viral replication (lack of CPEs), 1- viral CPEs in 25% of cells, 2 - viral CPEs in 50% of cells, 3 - viral CPEs in 75% of cells, and 4 - 100% of the cells affected with CPEs. Viral titer was expressed with reference to the TCID_50_. Positive controls were viruses incubated with 50% ethanol, negative controls were viruses incubated in DMEM 2% FBS (culture medium). The protocol for inactivation assay was prepared with reference to Tai et al. (2015).

### Statistical Analysis

Gompertz growth model *y* = *aexp*⁡(−*bexp*⁡(−*cx*)) and exponential model *y* = *aexp*⁡(*bx*) were fitted to estimate the concentration-dependent decrease in virus titer after treatment with EGb and its phytochemical components. Based on those models, *IC*_*50*_(inhibitory concentration) – concentration of the extract that inhibited 50% of viral replication when compared to the virus control – were calculated. Cytotoxicity of EGb was measured with luminescent and CTEs assay. Concentrations *CC*_*50*_ (cytotoxic concentration) – concentration of the extract that reduced the cell viability by 50% when compared to untreated control – were estimated based on fitted sigmoid curves $y=a+\frac{b-a}{1+\exp\left(\left(c-x\right)/d\right)}$. Differences in the kinetics of antiviral activity between EGb’ phytocomponents were measured with the following function *R*(*x*) = *E*(*activity*|*d*≥*x*), where *x* ∈ {6,15.5,15,20,23,25}μ*g*/*ml*, which is the expected value (i.e., mean) of antiviral activity if concentration *d* is equal or higher than *x*. Confidence intervals for a mean were estimated with bootstrap sampling. The difference between HHV-1 and HHV-2 concentration-dependent decrease in viral titer after treatment with EGb was tested with Hotelling’s *T*^2^statistic for vectors of Gompertz model parameters. Variance-covariance matrixes were estimated based on bootstrap sampling and degrees of freedom were computed with Nel and van der Merwe method. Selectivity index (SI) is a ratio that measures the window between cytotoxicity and antiviral activity. The higher the SI ratio, the more effective and safe a drug would be during *in vivo* treatment for a given viral infection (Pritchett et al., 2014). SI was calculated from the ratio CC_50_/IC_50_. Relative concentration *x* was computed as $R\,C\,\left(x\right)=\frac{x}{C\,C_{50}}$. Cohen’s γ was used as a measure of effect size for difference between two means.

## Results

### Assessment of Non-toxic Concentrations of EGb

**FIGURE 1:**
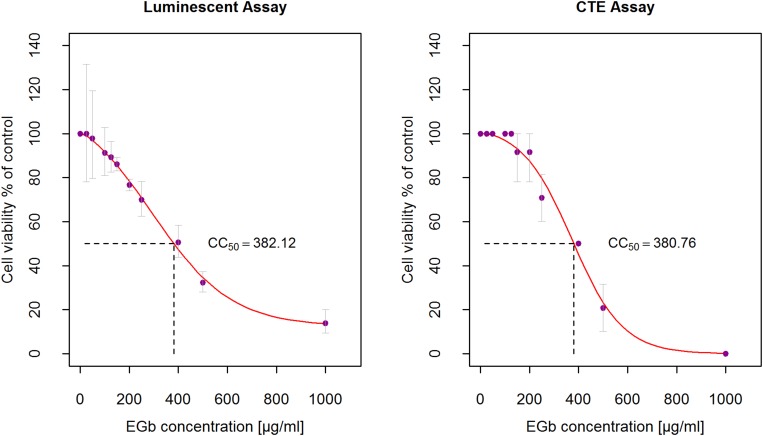
Determination of the 50% cytotoxic concentration (CC_50_) of EGb on A549. A549 cells were treated with serial dilutions of EGb (25–1000 μg/ml) and incubated for 72 h at 37°C/5% CO_2_. Cells viability were measured with the luminescent assay (*n* = 3) and CTEs assay (*n* = 6). CC_50_ was calculated based on fitted sigmoid curves. Error bars are CI95% of the means.

Starting solution of EGb 20 mg/ml in DMSO was diluted to final concentrations of 25–1000 μg/ml in DMEM 2% FBS. A549 cells were seeded in a 96-well plate (1.8 × 10^5^ cells/ml) and incubated 24 h at 37°C/5% CO_2_ incubator to obtain a monolayer. 24-h cultures monolayers of A549 cell with confluence > 90% were treated with serial dilutions of EGb and incubated for 72 h at 37°C/5% CO_2_. Morphological changes of the cells were observed every day under an inverted microscope and cell viability was calculated based on a CTEs scale. The final concentrations of DMSO < 2% were non-toxic. Results are presented in Supplementary Table S1 (Supplementary File-Table 2). Fresh EGb dilutions were prepared each time before starting a series of experiments. EGb in the range of 400 to 1000 μg/ml resulted in high cytotoxicity (CTEs = 3–4). Slight cytotoxic effects were observed for EGb in concentrations 200–250 μg/ml (CTEs = 1). EGb in the range of 25–150 μg/ml was non-toxic for A549 (CTEs = 0). EGb cytotoxicity, measured as a percent of cell viability in function of EGb concentration, was investigated also with luminescent assay for the same concentrations. Control were cells incubated only with culture medium DMEM 2% FBS. Figure 1 shows experimental measurements and sigmoid regression curves. Both curves has different slopes, however, estimated cytotoxic concentration of EGb which reduced viability of cells by 50% (CC_50_) was the same for two methods and equal to *CC*_50_≅381.5μ*g*/*ml* (average).

### Antiviral Activity of EGb

A potential antiviral effect of EGb against herpesviruses HHV-1 and HHV-2 was examined by early viral entry assay (inactivation assay). The test was performed to examine whether EGb could inactivate herpesviruses in cell-free state (free virus particles) and eventually prevent subsequent infection. EGb in the range of 25–500 μg/ml was incubated with HHV-1 and HHV-2 by 0.5 h (‘Short-term’), 1 and 2 h (‘Long-term’), and next titrated in A549 cells. Viral titer was calculated with reference to the TCID_50_. The highest dilution of the virus at which the CPEs occurred in about 50% of the cells was determined as 1 TCID_50_. Each variant of the experiment was performed three times in two independent repetitions each. Negative control were viruses incubated only with culture medium DMEM 2% FBS, and positive control were viruses incubated with 50% ethanol, which reduced virus infectivity of over 4 log (efficacy 99,99%). DMSO concentrations ≤ 2% did not inactivate HHV-1 and HHV-2. Results are presented in Supplementary Table S2 (Supplementary File-Table 2). EGb in a concentration-dependent manner appeared to interact with the cell-free virions, resulting in irreversible effects that protected the cell monolayer from the subsequent infection. These results suggested that EGb have a direct impact on these free virus particles by inactivating them and neutralizing their infectivity in non-toxic concentrations. EGb decline viral titer even of 4 log TCID_50_ which means 99.99% decrease of virus infectivity. With regards to the obtained results, a decrease in viral infectivity (viral titer), i.e., the effectiveness of EGb in inactivation of HHV-1 and HHV-2, was assessed. The ability of EGb to inactivate both viruses was observed already after 0.5 h and increased with the time of incubation (0.5 < 1 < 2 h). In addition, along with the increasing time of EGb incubation with both viruses, the lower concentrations of EGb exhibited prominent antiviral activity. The concentration of EGb which inhibited viral replication in 50% of cells (IC_50_, inhibitory concentration) was calculated on the basis of the time of incubation. In order to evaluate EGb antiviral activity (inactivation efficiency), a selectivity index (SI) was determined according to formula CC_50_/IC_50_. Results are presented in Table 1. Additionally, differences in pH of DMEM 2% FBS (medium for EGb dissolution) in selected solutions of EGb which were used in antiviral activity tests were investigated. It was showed that pH was constant during incubation time and did not influence on antiviral activity of EGb. Results are showed in Supplementary Table S3 (Supplementary File-Table 2).

**TABLE 1:** Antiviral activity of EGb against HHV-1 and HHV-2.

	**HHV-1**			**HHV-2**		
**Incubation time [h]**	**0.5**	**1**	**2**	**0.5**	**1**	**2**
CC_50_ [μg/ml]	381.5	381.5	381.5	381.5	381.5	381.5
IC_50_ [μg/ml]	79.1	68.2	29.6	137.3	45.5	8.1
SI	4.8	5.6	12.8	2.8	8.4	47.1

*CC_50_ (cytotoxic concentration) – concentration of the extract that reduced the cell viability by *50*% when compared to untreated control. IC_50_ (inhibitory concentration) - concentration of the extract that inhibited 50% of viral replication when compared to the virus control. SI (selectivity index) – calculated from the ratio CC_50_/IC_50_.*

### Antiviral Activity of *G. biloba* Flavonoids and Terpenes Compared to EGb

**FIGURE 2:**
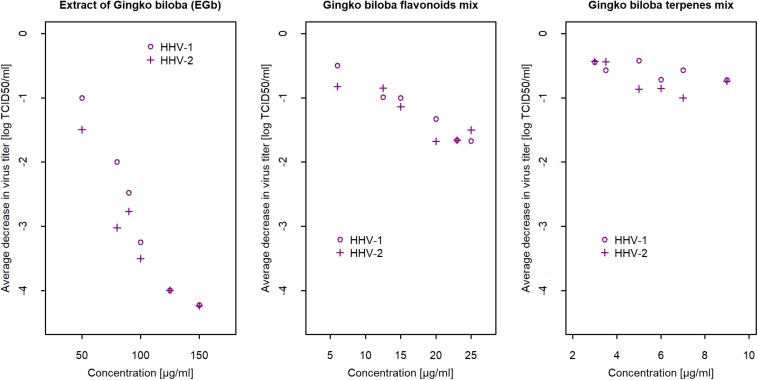
Mean reduction of HHV-1 and HHV-2 titer after 2 h of incubation with EGb, and with flavonoids mix and terpenes mix in relation to concentration (*n* = 6). Confidence intervals CI95% for means are presented in Supplementary Table S4 (Supplementary File-Table 2). Viral titer was calculated with reference to the TCID_50_. Reduction of viral titer was defined as difference in logs_10_ between concentration and control.

To establish if EGb presented higher antiviral effects than its phytochemical constituents and which fraction (*G. biloba* flavonoids or terpenes) is responsible for anti-herpesviral activity, a comparison of the preparations was performed. In all experiments, flavonoids mix (*G. biloba* flavonoids mix) and terpenes mix (*G. biloba* terpene lactones mix) were examined in concentrations consistent with the total amount in each EGb. Cytotoxicity was determined based on the CTEs scale. Maximum non-toxic concentrations for flavonoids was 25 μg/ml, whereas for terpenes 9 μg/ml (data not shown). Further analysis showed that the observed reduction of viral titer (for both viruses) by EGb was caused by flavonoids rather than terpenes. There was a clear concentration-dependence in HHV-1 as well as HHV-2 titer reduction for flavonoids mix, opposite to terpenes mix, where no relationship was observed and viral titer reduction was vestigial. Compounds were diluted in DMEM with 2% FBS. Negative control were viruses incubated only with culture medium DMEM 2% FBS, and positive control were viruses incubated with 50% ethanol, which reduced virus infectivity of over 4 log (efficacy 99,99%). The final concentrations of methanol ≤ 10% were non-toxic. Results are presented in Supplementary Table S1 (Supplementary File-Table 2). The maximal concentration of terpenes mix at 9 μg/ml caused an average reduction in viral titer respectively −0.72 logs for HHV-1 and −0.74 logs for HHV-2. Virus infectivity decreased $\frac{1}{18.6\times 10^{-2}}\approx 5$ times compared to controls. Figure 2 shows a concentration-dependent reduction in viral titer for EGb and its two constituents: flavonoids mix and terpene mix. Interestingly, as shown in Figure 2 and in Supplementary Table S4, EGb exhibited higher antiviral activity than flavonoids and terpenes. Supplementary Table S4 (Supplementary File-Table 2) contains the results of average changes in HHV-1 and HHV-2 titer after 2 h of incubation with EGb, flavonoids mix and terpene mix. The results clearly showed that there was a strong effect of EGb. EGb concentration equal to 80 μg/ml reduced HHV-1 average titer of −2 logs, i.e., 100 times, but virus titer reduction for 150 μg/ml was *log*_10_(−4.23)→10^−4.23^ = 0.000059→*reduction*:−99.9941%, i.e., the activity was $\frac{1}{5.9\times 10^{-5}}=16949$ times lower compared to control (*c* = 0μ*g*/*ml*). Similar relation was observed for EGb and HHV-2. Methanol concentrations ≤ 25% did not inactivate HHV-1 and HHV-2. Results are presented in Supplementary Table S2 (Supplementary File-Table 2).

### Comparison of Antiviral Action of Isorhamnetin, Kaempferol, and Quercetin

**FIGURE 3:**
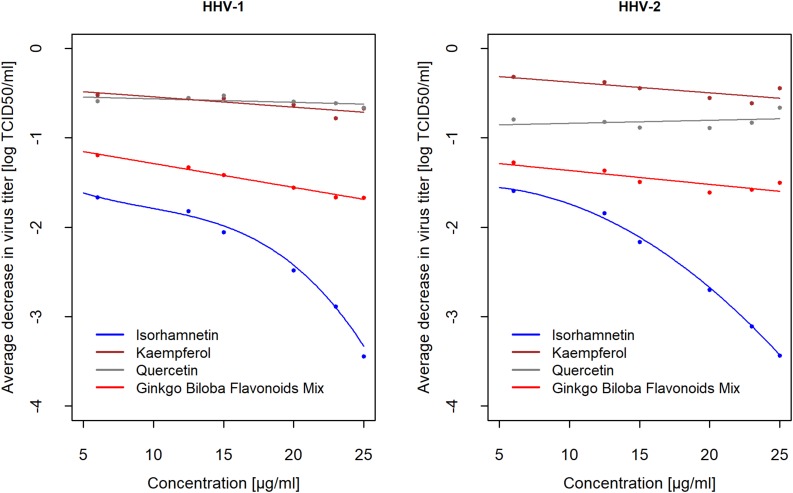
Average decrease of HHV-1 and HHV-2 titer (in *log*_*10*_) after 2 h of incubation with flavonoids mix, isorhamnetin, kaempferol, and quercetin compared to controls in relation to concentrations (*n* = 6). Average difference *treatment-control* was showed as the mean of decrease *ifconcentration*≥*x*, *wherex* ∈ {6,12.5,15,20,23,25}μ*g*/*ml*. Confidence intervals CI95% for averages are presented in Supplementary Table S5 (Supplementary File-Table 2). Viral titer was calculated with reference to the TCID_50_. Reduction of viral titer was defined as difference in logs_10_ between concentration and control.

Based on the results presented above, it was established that antiviral activity of EGb probably depends mostly on flavonoids. It was justified, therefore, to determine if particular flavonoids may express anti-herpesviral activity. Supplementary Table S5 (Supplementary File-Table 2) presents the mean change in virus titer after incubation with flavonoids mix and particular flavonoids, i.e., isorhamnetin, kaempferol, and quercetin. The results of the analysis showed a strong concentration-dependent reduction of HHV-1 and HHV-2 titer only for isorhamnetin. For concentration *d* = 5μ*g*/*ml* HHV-1 titer decreased by *log*_10_(−3.44)→10^−3.44^ = 0.000363→*reduction*:−99.964%, i.e., the activity was $\frac{1}{3.63\times 10^{-4}}=2754$ times lower compared to concentration *d* = 0μ*g*/*ml* (control). On the contrary, at a concentration of *d* = 6μ*g*/*ml* HHV-1 titer decreased *log*_10_(−0.89)→10^−0.89^ = 0.13→*reduction*:−87.11%, i.e., the activity was only $\frac{1}{1.29\times 10^{-1}}≅8$ times lower compared to concentration *d* = 0μ*g*/*ml* (control). Similar results were obtained for HHV-2. There was no concentration-dependent effect for kaempferol and quercetin. The reduction of HHV-1 and HHV-2 titer was smaller for the highest concentrations, and smaller than the reduction of viral titer by isorhamnetin at the smallest concentration. Flavonoids mix inactivated HHV-1 and HHV-2 much more than kaempferol and quercetin, however much less than isorhamnetin (Figure 3). Compounds were diluted in DMEM with 2% FBS. In those experiments negative control were viruses incubated only with culture medium DMEM 2% FBS, and positive control were viruses incubated with 50% ethanol, which reduced virus infectivity of over 4 log (efficacy 99,99%).

To present the difference in the kinetics of antiviral action for izorhamnetin, kaempferol and quercetin, let introduce the function *R*(*x*) = *E*[*activity*|*d*≥*x*], *x* ∈ {6,12.5,15,20,23,25}μ*g*/*ml*, which is the expected value (i.e., mean) reduction of HHV-1 and HHV-2 titer if concentration *d* is equal or higher than *x*. The results, calculated for every concentration based on data from Supplementary Table S5 (Supplementary File-Table 2), are presented on Figure 3. For example, the average decrease for *x* = 6μ*g*/*ml* is the mean difference for concentrations ≥6, i.e., simply, it is the general mean for all experiments. The second point is the mean difference for concentrations ≥12.5 and so on. The last point is the mean of decrease at a higher concentration *x* = 25μ*g*/*ml*. Results for both viruses are similar. As mentioned above, among three flavonoids only isorhamnetin decreased HHV-1 and HHV-2 titer and concentration-dependent decreasing had a quadratic relationship. For isorhamnetin concentration *x* = 25μ*g*/*ml* confidence interval for mean decrease of HHV-1 titer was CI95% (−3.89; −3), certainly a population (unknown) decrease is at least 1,000 times and no higher than 7,700 times compared to control. For HHV-2, these effects were similar, i.e., CI95% (−3.78; −3.11).

### Efficiency of EGb and Isorhamnetin in Impeding of HHV-1 and HHV-2 Primary Infection

**FIGURE 4:**
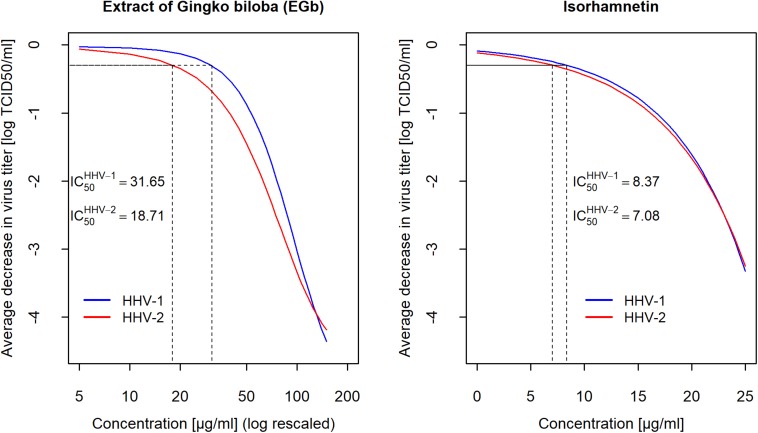
Fitted curves estimated for concentration-dependent decreasing of for HHV-1 and HHV-2 titers after 2 h of incubation with EGb and isorhamnetin. Gompertz growth model and exponential model was fitted for EGb and isorhamnetin, respectively. The marks on the x-axis on left graph increase logarithmically. Viral titer was calculated with reference to the TCID_50_.

A comparative analysis was also performed for evaluation of antiviral activity of EGb and isorhamnetin, the flavonoid that expressed the highest anti-herpesviral action. Cytotoxicity of isorhamnetin was estimated based on CTEs assay. CC_50_ for isorhamnetin was $C\,C_{50}^{I\,s\,o}=61.85\,\mu \,g/m\,l$. To compare the effectiveness of EGb and isorhamnetin in the inactivation of HHV-1 and HHV-2, two independent conditions must be met. First, any considered concentrations had to correspond to 100% cells viability, and second, concentrations expressed relative to CC_50_ had to be the same for EGb and isorhamnetin. Based on cytotoxicity results for EGb and isorhamnetin in CTEs assay, the highest accepted concentration for EGb equal to 125μ*g*/*ml* (Figure 1) was taken. It gave the relative concentration $R\,C^{E\,G\,b}=\frac{125}{381.5}=0.327$. Based on these results, the highest accepted concentration for isorhamnetin could not be higher than $0.327\times \left(CC_{50}^{I\,s\,o}=61.85\right)=20.3\mu g/ml$. The concentration of isorhamnetin equal to 20μ*g*/*ml* manifested in 100% cells viability, so we had $R\,C^{I\,s\,o}=\frac{20}{61.85}=0.323$. Results presented in Supplementary Tables S4, S5 (Supplementary File-Table 2) clearly show that for the same relative concentrations, EGb caused a reduction of HHV-1 and HHV-2 titer by −3.99*log*TCID_50_, which is much higher compared to isorhamnetin. Flavonoid reduced HHV-1 titer about −1.67*log* TCID_50_ and HHV-2 about −1.89*log* TCID_5__0_ respectively. Isorhamnetin in a concentration of 25μ*g*/*ml* reduced viral titer about −3.44*log* TCID_50_ for both viruses and this is 0.4 of $C\,C_{50}^{I\,s\,o}$ (i.e., closer to*CC*_*50*_), but still the titer reduction is about 6 times weaker compared to EGb in concentration equal to 125μ*g*/*ml*. Based on the data presented in Supplementary Tables S4, S5 (Supplementary File-Table 2), the estimated $I\,C_{50\,\left(I\,s\,o\right)}^{H\,H\,V-1}=8.37\,\mu \,g/m\,l$, $I\,C_{50\,\left(I\,s\,o\right)}^{H\,H\,V-2}=7.08\,\mu \,g/m\,l$ for isorhamnetin, and $I\,C_{50\,\left(E\,G\,b\right)}^{H\,H\,V-1}=31.65\,\mu \,g/m\,l$, $I\,C_{50\,\left(E\,G\,b\right)}^{H\,H\,V-2}=18.71\,\mu \,g/m\,l$ for EGb. Results are shown in Table 2. Confidence intervals CI95% for IC_50_ were *CI*95(20.7,42.8) and *CI*95(10.3,29.4) for HHV-1 and HHV-2, respectively. Difference $I\,C_{50\,\left(E\,G\,b\right)}^{H\,H\,V-1}-I\,C_{50\,\left(E\,G\,b\right)}^{H\,H\,V-2}=12.94\,\mu \,g/m\,l$ with confidence interval *CI*95(−3.5;29.4), statistic *z* = 1.54 for this difference and *p* = 0.1238, so there was no proof that IC_50_ for both HHV-1 and HHV-2 was different. The precision of estimation of this difference was measured with Cohen’s *γ* effect-size and gave the result γ = 2.18 with [CI95(2.13, 2.21)]; therefore, according to Sawilowsky’s interpretation, this effect was huge (Shlomo, 2009). Figure 4 shows a concentration-dependent reduction of HHV-1 and HHV-2 titer after 2 h of incubation with EGb and isorhamnetin. We can see that fitted curves for EGb differ between HHV-1 and HHV-2; however, such difference is not obtained for isorhamnetin. Our next questions naturally involved whether two curves for EGb statistically differ or not. Hotelling’s *T*^2^ statistic for vectors of the parameters of the two curves is $T^{2}=3.86∼\chi_{d\,f=1.9}^{2}\to p=0.0494$. We can conclude that probably kinetic of EGb activity differs for HHV-1 and HHV-2. Trace of this difference can be seen also in Figure 3. In addition, HHV-2 was more sensitive to quercetin than HHV-1. However, we cannot reject the hypothesis that there were interactions between particular EGb′ constituents and the general effect of EGb was not a sum of effects of its phytochemical constituents.

**TABLE 2:** Effectiveness of EGb and isorhamnetin in inactivation of HHV-1 and HHV-2.

**Compound**	**Concentration**	**CC_50_**	**Relative concentration**	**Cell viability**	**Mean of viral**		**IC_50_**	
	**[μg/ml]**		**(% of CC_50_)**	**(CTEs assay)**	**titer reduction**			
					**HHV-1**	**HHV-2**	**HHV-1**	**HHV-2**
EGb	125	381.5	32.7	100%	−3.99	−3.99	31.65	18.71
Isorhamnetin	20	61.85	32.3	100%	−1.67	−1.89	8.37	7.08

## Discussion

An awareness of the potential opportunities in new therapies for herpesviruses HHV-1 and HHV-2 infections has developed. Some drugs for the management of herpesviruses infections, such as acyclovir, are currently available. However, due to the prevalence of resistance in acyclovir-treated immunocompromised individuals, there is a need to search for new preparations with anti-herpes activity. The overarching goal of developing novel prophylactics or therapeutics against HHV-1 and HHV-2 is to prevent the initial infection or viral cell-to-cell spread. Plant extracts offer safe platforms for the discovery of efficient and novel antiviral agents. One of the promising plant-derived anti-herpesviral agent – extract of *G. biloba* (EGb) – has been summarized in this paper. In the light of obtained results, EGb could prove a valuable prophylactic and therapeutic in humans.

The current state of research suffers deficiencies in studies demonstrating antiviral activity of EGb against herpesviruses such as HHV-1 and HHV-2. Limited investigations showed anti-human influenza virus or anti-HIV activity of *G. biloba* preparations. However, this data does not always refer to the dry leaves extract, but rather to other constituents obtained from *G. biloba*, such as polyphenols or organic acids as mentioned above. Importantly, depending on the plant origin and method of extraction, the final products can differ significantly in their activity (Kressmann et al., 2002). In addition, authors who present the antiviral activity of EGb or other *G. biloga* constituents frequently derive the preparations using small-scale laboratory methods. It is difficult then to establish standardization. This problem also refers to other botanical extracts with potential anti-herpesviruses activity, e.g., hydroalcoholic extract prepared from roots of *Hemidesmus indicus* (Bonvicini et al., 2018). The extract, however, exhibited high anti-HHV-1 and anti-HHV-2 activity at non-cytotoxic concentrations. Additionally, it should be underlined that EGb patent has expired and many pharmaceutical companies produce preparations that differ from each other in composition and therapeutic properties (Isah, 2015). The first benefit of EGb investigated in this study is its accessibility and composition. This is a standardized extract, prepared according to the European Pharmacopoeia (Ph. Eur. 8.0) [see section “EGb Characterization” (Supplementary File-Table 1)].

Preparations with valuable antiviral activity must inhibit the growth of a virus without exerting toxicity on the infected cells. We used the microscopic method, CTEs and luminescence method to determine the non-toxic concentrations of EGb before commencing with antiviral activity investigations. With regards to adherent lines, cells that have separated from the surface may still be metabolically active but recognized as undergoing CTEs. In the luminescence test, therefore, we could obtain higher cell viability. Although the CTEs method is more rigorous compared to other cellular tests, in our calculations the estimated CC_50_ was the same for two methods (average was 381.5 μg/ml). EGb was non-toxic for living cells up to 150 ug/ml, only slight toxic effects were observed for concentrations ranging from 200 to 250 μg/ml. Importantly, all non-toxic concentrations of EGb showed prominent antiviral activity against HHV-1 and HHV-2, which is the second benefit of this extract. Antiviral activity of other plant-derived compounds, such as biflavone ginkgetin from *Cephalotaxus drupacea*, against herpesviruses have been previously reported (Hayashi et al., 1992). There are, however, some limitations in the use of such preparations due to their action across a broad range of sub-cytotoxic concentrations.

Since viral entry into target cell is an important step in the viral replicative cycle, blocking this mechanism can lead to the suppression of HHV-1 and HHV-2 infectivity and provide novel antiviral strategy. Herpesvirus entry into the cell is a complex process that starts with the attachment of positively charged viral glycoproteins gB and/or gC with negatively charged HS proteoglycans (HSPG) present on the cell surface. Viral nucleocapsid is next transported to the nucleus where viral DNA is replicated (Agelidis and Shukla, 2015). Both herpesviruses were significantly inhibited by pretreatment with EGb prior to the infection of cells, which indicates that EGb affected the virus before adsorption to cell surface. We have shown that EGb is highly effective in diminishing the infectivity of both HHV-1 and HHV-2 in *in vitro* models of infection by direct inactivation of virions. Even by 4 log TCID_50_ reduction of both viruses titer with 125 ug/ml EGb was observed, which means a 99.99% decrease in infectivity. Interestingly, EGb strongly inactivated HHV-1 just after 30 min of incubation. Along with the time of incubation, the capacity of EGb to inactivate HHV-1 and HHV-2 increased. This mechanism – viral particles trapping – lets us hamper the initial infection which in turn may prevent cell-to-cell spread. Possibly, the mechanism of EGb action might be similar as those recently discovered for zinc oxide tetrapods (Mishra et al., 2011; Antoine et al., 2012). It has been revealed that this nanostructures can bind up positively charged viral glycoproteins involved in attaching to cell surface, thus decreasing cell–virus interactions. Another possible mechanisms are interference in envelope, proteins and/or genome structure of the virus. Maybe the EGb action lead to render the genome non-replicable or inhibit host-cell recognition/binding. Modifications in the virus proteins or genome may lead to the inhibition of these functions and eventually inactivation viral particles. These mechanisms are characteristics for disinfectants (Wigginton et al., 2012). It should be noticed, however, that disinfectants act often in high toxic concentrations, whereas EGb inactivate viruses in non-toxic concentrations. Future experiments to confirm this mechanism for EGb should be carried out.

We have investigated antiviral activity of EGb against different viruses belonging to different taxonomic groups, i.e., VSV – *Vesicular stomatitis virus*; Indiana strain; ATTC VR-1238^TM^, *Rabdoviridae (enveloped, RNA);* ECBO – *Enteric cytopathogenic bovine orphan virus*; LCR-4 strain; ATTC VR-248^TM^, *Picornaviridae (non-enveloped, RNA);* HAdV-5 – *Human adenowirus 5*; Adenoid 75 strain; ATTC VR-5^TM^, *Adenoviridae (non-enveloped, DNA*). To investigate influence of EGb on viruses replication steps (i.e., handicap viruses adsorption to the cells surface by blocking appropriate receptors, block out activity of replication enzymes, induction of antiviral conditions in the cells, impeding release of free viral particles from the cells) three antiviral assays (AVA) have been carried out: cell incubation with EGb before viruses adsorption, cell incubation with viruses and EGb simultaneously, and cell incubation with EGb after viruses adsorption. Direct antiviral effect – inactivation of free viral particles was also investigated. EGb did not express antiviral activity against none of these viruses. It should be underlined, however, that among tested viruses strong antiviral activity of EGb were observed only for herpesviruses HHV-1 and HHV-2 (DNA, enveloped). Admittedly EGb did not act before and after HHV-1 and HHV-2 penetration into the host cell [data presented in Supplementary Table S6 (Supplementary File-Table 2)], the extract, however, was capable of exerting a direct antiviral effect on herpesviruses free particles. The high reduction of HHV-1 and HHV-2 titer by EGb is another benefit of the extract compared to the other extracts with anti-herpesviral activity. Direct treatment of HHV-1 with *Mentha pulegium* extract was shown to resulted in 1.7 log TCID_50_ reduction in virus titers after 1 h (Parsania et al., 2017). Nevertheless, an effect similar to that of EGb was obtained for *Melissa officinalis* essential oil (Schnitzler et al., 2008). Melissa oil was capable of exerting a direct antiviral effect on herpesviruses. It affected the virus before adsorption, but not after penetration into the host cell. Melissa oil reduced HHV-1 and HHV-2 titer by 98.8 and 97.2%, respectively.

Our next questions naturally involved which phytochemical constituents of EGb -flavonoids or terpenes- were responsible for the anti-herpesviral activity of EGb. Flavonoids and terpene lactones from *G. biloba* are the primary compounds that are extensively studied due to their variety of pharmacological activities. Based on the results from inactivation assay, we showed that flavonoids rather than terpene lactones were responsible for anti-HHV-1 and anti-HHV-2 activity. Nevertheless, the observed antiviral activity (inactivation of free viruses particles) was lower for flavonoids mix and terpenes mix than for EGb. The tested *G. biloba* flavonoids mix contained the three most important flavonols, i.e., isorhamnetin, kaempferol, and quercetin. Next, we investigated the antiviral activity of these three compounds. We had expected the antiviral activity of flavonoids fraction against herpesviruses due to previously described antiviral effects of different plant-derived flavonoids. Anti-HHV-1 effects of quercetin and kaempferol from *Ficus benjamina* leaves has been previously reported (Yarmolinsky et al., 2012). However, we did not confirm such activity for quercetin and kaempferol from *G. biloba.* Surprisingly, prominent antiviral effect was noticed for isorhamnetin. We showed that isorhamnetin has a strong ability to inactivate HHV-1 and HHV-2. Biological activities of isorhamnetin, such as antioxidant, anticancer, antimicrobial, and anti-inflammatory effects, are well described (Kandakumar and Manju, 2017). Studies of antiviral activity, however, are limited. Isorhamnetin showed antiviral effect against influenza virus. It has been established in both *in vitro* and *in vivo* studies that isorhamnetin acts via direct hemagglutinin (HA) and neuraminidase (NA) inhibition, and direct or indirect inhibition of the expression of viral HA and NA gene (Abdal Dayem et al., 2015). Several other flavonoids have been described for their antiviral activity (Kumar and Pandey, 2013). It is expected that, because flavonoids are widely distributed in plants, they should express minimal toxicity. Nevertheless, these compounds have a diverse range of activities for living cells. Antiviral activity but with high cytotoxicity can limit the practical usefulness of most flavonoids and plant extracts presented in scientific literature.

The results obtained with isorhamnetin and EGb, however, raised the question which of these preparations is more effective in diminishing herpes viruses infectivity. This prompted us to compare the antiviral activity of these two preparations. Analysis revealed interesting results showing that anti-HHV-1 and anti-HHV-2 activity of EGb was much higher compared to isorhamnetin. Moreover, EGb had lower toxicity. These EGb′ features allow it to be considered as more promising in the treatment of HHV-1 and HHV-2 infections. The study, however, has one limitation. In regards to the lack of significant anti-herpesviral activity of terpenes mix, each component’s antiviral effects, i.e., bilobalid, ginkgolide A, ginkgolide B, ginkgolide C, ginkgolide J, have not been analyzed. However, it was revealed that flavonoids are the main fraction responsible for anti-herpesviral activity, with the highest activity for isorhamnetin. Importantly, it should be underlined that EGb is a complex compound that consists of several components. Possibly, combined effects of its constituents decide about strong anti HHV-1 and anti-HHV-2 activity.

The potential use of EGb in multidrug therapy with synthetic anti-herpes compounds can be considered. The genetic diversity of herpesvirus genomes within an infected individual is now well-established, and this is often related to resistance to current antiviral drugs. Eventually, EGb may be prospectively considered and implemented in combination therapy with standard antivirals when the suppression of viral replication provided by this preparations is not complete, and cannot be supported by the immune system in immunocompromised hosts or in conditions where the immune system is thought to be a key factor in the development of the disease. This *in vitro* studies showed very interesting activity of EGb and its phytochemical constituents. Future *in vivo* investigations, however, are needed to confirm EGb efficacy. Latent HHV-1 in the brain may be reactivated intermittently by events such as immunosuppression, peripheral infection, and inflammation, the consequent accumulating damage culminates eventually in the development of AD. It is true that, at the moment, there is not enough evidence to know whether treating infections would be a good strategy to treat AD or to reduce the risk of the disease. This is why further research is needed to shed more light on this important topic.

## Conclusion

In summary, standardized EGb shows high anti-HHV-1 and anti-HHV-2 activity in non-toxic concentrations and significantly reduces the infectivity of both pathogens. Most likely, EGb could augment current therapies for herpes labialis and genital herpes, especially in the treatment of skin ailments during recurrent infections. A combination of antiviral agents with different molecular targets, including EGb, has the potential to keep HHV-1 and HHV-2 replication to a minimum.

## Data Availability Statement

All datasets generated for this study are included in the manuscript/Supplementary Files.

## Author Contributions

MarS and JL: conceptualization, funding acquisition, project administration, and supervision. MarS, MacS, and MO: data curation. MarS and MacS: formal analysis and visualization. MO, KZ, and MarS: investigations. MarS, MO, and KZ: methodology. MarS, MacS, and JL: writing – original draft, review, and editing.

## Conflict of Interest

The authors declare that the research was conducted in the absence of any commercial or financial relationships that could be construed as a potential conflict of interest.

## Abbreviations

CNS: central nervous system
CC_50_: cytotoxic concentration
CI: confident interval
CPEs: cytopathic effects
CTEs: cytotoxic effects
DMEM: Dulbecco medium
DMSO: dimethyl sulfoxide
EGb: extract of *Ginkgo biloba*
FBS: fetal bovine serum
HA: hemagglutinin
HHV-1: human alphaherpesvirus 1
HHV-2: human alphaherpesvirus 2
HIV: *human immunodeficiency virus*
IC_50_: inhibitory concentration
NA: neuraminidase
PEDV: *porcine epidemic diarrhea virus*
SI: selectivity index
TCID_50_: tissue culture infectious dose.
