# Supplementary material for: Hampering Herpesviruses HHV-1 and HHV-2 Infection by Extract of Ginkgo biloba (EGb) and Its Phytochemical Constituents
Source: Front Microbiol. 2019 Oct 15;10:2367. doi: 10.3389/fmicb.2019.02367 (PMC6803450; doi:10.3389/fmicb.2019.02367)
Supplement: Supplementary file 1 [file Table_1.DOCX]

**EGb CHARACTERIZATION**

**Extract of *G. biloba* (EGb)**

Dry extract from *G. biloba* leaf (GINKGONIS EXTRACTUM SICCUM RAFFINATUM ET QUANTIFICATUM PH. EUR. [[European Pharmacopoeia](https://www.google.pl/url?sa=t&rct=j&q=&esrc=s&source=web&cd=1&cad=rja&uact=8&ved=2ahUKEwid67WP2KHhAhVqmIsKHVAQDX4QFjAAegQIABAB&url=https%3A%2F%2Fwww.edqm.eu%2Fen%2Feuropean-pharmacopoeia-ph-eur-9th-edition&usg=AOvVaw1azj1VeC3PPlZk-pi5Y-EX)]) (Martin Bauer Group, Finzelberg GmbH & Co. KG, Germany).

*Detailed characteristic:* Dry extract from G. biloba leaf without excipients, batch No. 16000375; date of production 18-05-2016.

*Quality data:* Characters: bright yellow-brown, powder; Particle size: powder (min 95%< 0,315 mm); Sieve>0,315 mm: 0,2% (max. 5); Bottom of sieve: 99,8% (min. 95); Loss on drying acc. to Ph. Eur. (2.8.17): 2,4 % (max 5); Bulk density: 410 g/l; Residual solvent 1-butanol (GC) acc. to Ph. Eur. (2.4.24): 0,0042 % (max.0,5); Residual solvent acetone (GC) acc. to Ph. Eur. (2.4.24): 0,0020 % (max.0,5); Residual solvent ethanol (GC) acc. to Ph. Eur. (2.4.24): 0,2449 % (max.0,5); Residual solvent 2-butanone (GC) acc. to Ph. Eur. (2.4.24): 0 (max.0,5); Residual solvent heptane (GC) acc. to Ph. Eur. (2.4.24): 0,0009 % (max.0,5); Identity TLC-Fingerprint on flavonoids acc. to Ph. Eur. (monograph Ginkgo dry extract, refined and quantified): complies; Assay flavonoids (HPLC) expressed as flavone glycosides (dried extract) acc. to Ph. Eur. (monograph Ginkgo dry extract, refined and quantified): 25.3 % (Specification: 22.0 % – 27.0 %); Assay ginkgolides A, B and C (HPLC) (dried extracts) acc. to Ph. Eur. (monograph Ginkgo dry extract, refined and quantified): 3.1 % (Specification: 2.8 % – 3.4 %); Assay bilobalide (HPLC) (dried extract) acc. to Ph. Eur. (monograph Ginkgo dry extract, refined and quantified): 2.8 % (Specification: 2.6 % – 3.2 %); Assay ginkgolic acids (HPLC) (dired extract) acc. to Ph. Eur. (monograph Ginkgo dry extract refined and quantified): 1.1 ppm (max. 5 ppm); Microbiological examination acc. to Ph. Eur. (2.6.31): complies (Specification: has to comply with Ph. Eur. (5.1.8 B)); Total aerobic microbial count (TAMC): <10 cfu/g (max. 50000 cfu/g); Total combined yeasts/moulds count (TYMC): <10 cfu/g (max. 500 cfu/g); Bile-tolerant Gram-negative bacteria: <1 cfu/g (max. 100 cfu/g); Escherichia coli/g: complies (Specification: absent); Salmonella/25 g: complies (Specification: absent). Storage: dry place, room temperature, dark bottle. Solvent: DMSO (Sigma-Aldrich, Poland). Certificate of analysis was authorized by the producer.
