# Supplementary material for: Hampering Herpesviruses HHV-1 and HHV-2 Infection by Extract of Ginkgo biloba (EGb) and Its Phytochemical Constituents
Source: Front Microbiol. 2019 Oct 15;10:2367. doi: 10.3389/fmicb.2019.02367 (PMC6803450; doi:10.3389/fmicb.2019.02367)
Supplement: Supplementary file 2 [file Table_2.DOCX]

**TABLES**

**Table S1** Cytotoxicity of DMSO and methanol on A549 cells (n=3)

| **DMSO [%]** | **CTE** | **methanol [%]** | **CTE** |
| --- | --- | --- | --- |
| **5** | **4** | 20 | 4 |
| **2,5** | **3** | 15 | 4 |
| **2** | **2** | 12,5 | 4 |
| **1,25** | **0** | 10 | 0 |
| **1** | **0** | 7 | 0 |
| **0,75** | **0** | 5 | 0 |
| **0,5** | **0** | 3,2 | 0 |
| **0** | **0** | 0 | 0 |

CTE-cytotoxic effects; 0 - lack of cytotoxic effects, 1- CTE in 25% of cells, 2 - CTE in 50% of cells, 3 - CTE in 75% of cells, and 4 – 100% of the cells affected with CTE

**Table S2** Inactivation of HHV-1 and HHV-2 with DMSO and methanol (n=3)

| **DMSO [%]** | **Average viral titer (log TCID50/ml)** | | **methanol [%]** | **Average viral titer (log TCID50/ml)** | |
| --- | --- | --- | --- | --- | --- |
|  | **HHV-1** | **HHV-2** |  | **HHV-1** | **HHV-2** |
| **2** | 5 | 5 | **50** | 0 | 0 |
| **1,5** | 5 | 5 | **32** | 0 | 0 |
| **1** | 5 | 5 | **25** | 4,5 | 4,5 |
| **0,5** | 5 | 5 | **20** | 4,5 | 4,5 |
| **0,25** | 5 | 5 | **15** | 4,5 | 4,5 |
| **0** | 5 | 5 | **0** | 4,5 | 4,5 |

**Table S3** Differences in pH of DMEM 2% FBS (medium for EGb dissolution) in selected solutions of EGb (n=3)

| **Incubation time [h]**  **EGb [μg/ml]** | **0** | **0.5** | **1** | **2** |
| --- | --- | --- | --- | --- |
| **200** | 7,63 | 7,72 | 7,78 | 7,84 |
| **150** | 7,65 | 7,73 | 7,81 | 7,86 |
| **100** | 7,64 | 7,72 | 7,78 | 7,83 |
| **DMEM 2% FBS** | 7,58 | 7,66 | 7,72 | 7,78 |

**Table S4** Mean (and CI95% for the mean) reduction of HHV-1 and HHV-2 titer after 2h of incubation with EGb, flavonoids mix and terpenes mix, in relation to concentration. Number of repetitions for every single experiment was at least $n=6$. Confidence interval 95% estimated with bootstrap method. Reduction of viral titer was defined as difference in $\mathrm{logs}_{10}$ between concentration and control (concentration = 0 µg/ml)

| **HHV-1** | **EGb** | Concentration  [µg/ml] | **50** | | **80** | | **90** | | **100** | | **125** | | **150** | |
| --- | --- | --- | --- | --- | --- | --- | --- | --- | --- | --- | --- | --- | --- | --- |
|  |  | Mean | -1.00 | | -2.00 | | -2.48 | | -3.25 | | -3.99 | | -4.23 | |
|  |  | CI95% | -1.5 | -0.5 | -2.5 | -1.5 | -3 | -1.75 | -3.75 | -2.75 | -4.5 | -3.5 | -4.75 | -3.75 |
|  |  | | | | | | | | | | | | | |
|  | **Flavonoids mix** | Concentration  [µg/ml] | **6** | | **12.5** | | **15** | | **20** | | **23** | | **25** | |
|  |  | Mean | -0.50 | | -0.99 | | -1.00 | | -1.33 | | -1.66 | | -1.67 | |
|  |  | CI95% | -0.83 | -0.17 | -1.67 | -0.33 | -1.67 | -0.33 | -1.67 | -1.00 | -2.00 | -1.33 | -2.00 | -1.33 |
|  |  | | | | | | | | | | | | | |
|  | **Terpenes mix** | Concentration  [µg/ml] | **3** | | **3.5** | | **5** | | **6** | | **7** | | **9** | |
|  |  | Mean | -0.44 | | -0.57 | | -0.42 | | -0.72 | | -0.57 | | -0.72 | |
|  |  | CI95% | -0.83 | 0.00 | -1.17 | -0.17 | -1.00 | 0.00 | -1.17 | -0.33 | -1.00 | -0.17 | -1.17 | -0.17 |
|  | | | | | | | | | | | | | | |
| **HHV-2** | **EGb** | Concentration  [µg/ml] | **50** | | **80** | | **90** | | **100** | | **125** | | **150** | |
|  |  | Mean | -1.50 | | -3.02 | | -2.77 | | -3.50 | | -3.99 | | -4.24 | |
|  |  | CI95% | -2.25 | -1 | -3.5 | -2.5 | -3.75 | -2 | -4 | -3 | -4.5 | -3.5 | -4.75 | -3.75 |
|  |  | | | | | | | | | | | | | |
|  | **Flavonoids mix** | Concentration  [µg/ml] | **6** | | **12.5** | | **15** | | **20** | | **23** | | **25** | |
|  |  | Mean | -0.83 | | -0.85 | | -1.14 | | -1.68 | | -1.66 | | -1.50 | |
|  |  | CI95% | -1.33 | -0.33 | -1.33 | -0.33 | -1.83 | -0.50 | -2.00 | -1.33 | -2.00 | -1.33 | -1.83 | -1.17 |
|  |  | | | | | | | | | | | | | |
|  | **Terpenes mix** | Concentration  [µg/ml] | **3** | | **3.5** | | **5** | | **6** | | **7** | | **9** | |
|  |  | Mean | -0.44 | | -0.44 | | -0.86 | | -0.85 | | -1.00 | | -0.74 | |
|  |  | CI95% | -1.00 | 0.00 | -1.00 | 0.00 | -1.33 | -0.33 | -1.33 | -0.33 | -1.50 | -0.50 | -1.33 | -0.33 |

**Table S5** Mean (and CI95% for the mean) reduction of HHV-1 and HHV-2 titer after 2h of incubation with flavonoids mix, izorhamnetin, kaempferol and quercetin depend on concentration. Number of repetitions for every single experiment was at least $n=6$. Confidence interval 95% estimated with bootstrap method. Reduction of viral titer was defined as difference in $\mathrm{logs}_{10}$ between concentration and control (concentration = 0 µg/ml)

| **Virus** | **Compound** | Concentration  [µg/ml] | **6** | | **12.5** | | **15** | | **20** | | **23** | | **25** | |
| --- | --- | --- | --- | --- | --- | --- | --- | --- | --- | --- | --- | --- | --- | --- |
| **HHV-1** | **izorhamnetin** | Mean | -0.89 | | -0.89 | | -0.78 | | -1.67 | | -2.33 | | -3.44 | |
|  |  | CI95% | -1.00 | -0.67 | -1.00 | -0.67 | -1.00 | -0.44 | -2.00 | -1.33 | -2.67 | -2.00 | -3.89 | -3.00 |
|  |  | | | | | | | | | | | | | |
|  | **kaempferol** | Mean | -0.34 | | -0.55 | | -0.34 | | -0.34 | | -0.89 | | -0.67 | |
|  |  | CI95% | -0.67 | -0.11 | -0.89 | -0.22 | -0.67 | -0.11 | -0.67 | 0.00 | -1.00 | -0.67 | -0.89 | -0.33 |
|  |  | | | | | | | | | | | | | |
|  | **quercetin** | Mean | -0.78 | | -0.66 | | -0.33 | | -0.56 | | -0.57 | | -0.66 | |
|  |  | CI95% | -1.00 | -0.44 | -0.89 | -0.33 | -0.67 | 0.00 | -0.89 | -0.22 | -0.89 | -0.22 | -1.00 | -0.33 |
|  |  | | | | | | | | | | | | | |
|  | **Flavonoids Mix** | Mean | -0.50 | | -0.99 | | -1.00 | | -1.33 | | -1.66 | | -1.67 | |
|  |  | CI95% | -0.83 | -0.17 | -1.67 | -0.33 | -1.67 | -0.33 | -1.67 | -1.00 | -2.00 | -1.33 | -2.00 | -1.33 |
|  |  | | | | | | | | | | | | | |
| **HHV-2** | **izorhamnetin** | Mean | -0.34 | | -0.55 | | -0.55 | | -1.89 | | -2.78 | | -3.44 | |
|  |  | CI95% | -0.67 | -0.11 | -0.89 | -0.22 | -0.89 | -0.22 | -2.22 | -1.44 | -3.00 | -2.55 | -3.78 | -3.11 |
|  |  | | | | | | | | | | | | | |
|  | **kaempferol** | Mean | 0.00 | | -0.11 | | -0.11 | | -0.44 | | -0.78 | | -0.45 | |
|  |  | CI95% | 0.00 | 0.00 | -0.33 | 0.00 | -0.33 | 0.00 | -0.78 | -0.11 | -1.00 | -0.44 | -0.78 | -0.11 |
|  |  | | | | | | | | | | | | | |
|  | **quercetin** | Mean | -0.67 | | -0.55 | | -0.89 | | -1.00 | | -1.00 | | -0.66 | |
|  |  | CI95% | -1.00 | -0.33 | -0.89 | -0.22 | -1.00 | -0.67 | -1.00 | -1.00 | -1.00 | -1.00 | -0.89 | -0.33 |
|  |  | | | | | | | | | | | | | |
|  | **Flavonoids Mix** | Mean | -0.83 | | -0.85 | | -1.14 | | -1.68 | | -1.66 | | -1.50 | |
|  |  | CI95% | -1.33 | -0.33 | -1.33 | -0.33 | -1.83 | -0.50 | -2.00 | -1.33 | -2.00 | -1.33 | -1.83 | -1.17 |

**Table S6** Antiviral activity of EGb against different viruses: VSV, HAdV-5, ECBO, HHV-1 and HHV-2 (n=4)

|  | **Cell incubation with EGb before viruses adsorption** | | | **Cell incubation with viruses and EGb simultaneously** | | | **Cell incubation after viruses adsorption** | | | **Direct antiviral activity (inactivation)** | | | |
| --- | --- | --- | --- | --- | --- | --- | --- | --- | --- | --- | --- | --- | --- |
|  | **EGb [μg/ml]** | | | | | | | | | | | | |
|  | **150** | **100** | **0/virus control** | **150** | **100** | **0/virus control** | **150** | **100** | **0/virus control** | **150** | **100** | **0/virus control** |  |
| **Viruses** | **Average viral titer [logTCID50/ml]** | | | | | | | | | | | |  |
| **VSV** | 5,3 | 5,5 | 5,0 | 5,0 | 5,0 | 5,0 | 4,5 | 5,0 | 4,5 | 4,5 | 5,3 | 5,5 |  |
| **HAdV-5** | 5,5 | 5,3 | 5,0 | 5,0 | 4,5 | 4,5 | 4,5 | 4,5 | 5,0 | 4,0 | 4,0 | 4,5 |  |
| **ECBO** | 3,5 | 3,5 | 3,5 | 3,8 | 4,0 | 4,0 | 3,5 | 3,8 | 3,5 | 3,8 | 3,8 | 3,5 |  |
| **HHV-1** | 6,25 | 5,75 | 5,5 | 5,0 | 5,25 | 5,5 | 5,5 | 5,5 | 5,0 | Data presented in the manuscript | | |  |
| **HHV-2** | 6,25 | 6,75 | 5,5 | 5,5 | 5,0 | 5,5 | 5,5 | 5,25 | 5,5 |  |  |  |  |
